# *Leishmania donovani* 90 kD Heat Shock Protein – Impact of Phosphosites on Parasite Fitness, Infectivity and Casein Kinase Affinity

**DOI:** 10.1038/s41598-019-41640-0

**Published:** 2019-03-25

**Authors:** Antje Hombach-Barrigah, Katharina Bartsch, Despina Smirlis, Heidi Rosenqvist, Andrea MacDonald, Florent Dingli, Damarys Loew, Gerald F. Späth, Najma Rachidi, Martin Wiese, Joachim Clos

**Affiliations:** 1 Bernhard Nocht Institute for Tropical Medicine, Hamburg, Germany; 2 Institut Pasteur and Institut National de Santé et Recherche Médicale INSERM U1201, Unité de Parasitologie Moléculaire et Signalisation, Paris, France; 3 Strathclyde Institute of Pharmacy and Biomedical Sciences (SIPBS) University of Strathclyde, Glasgow, Scotland UK; 4 Hellenic Pasteur Institute, Athens, Greece; 5 Laboratoire de Spectrométrie de Masse Protéomique, Centre de Recherche, Institut Curie, PSL Research University, Paris, France; 6 Present Address: Novo Nordisk A/S, Gentofte, Denmark

## Abstract

*Leishmania* parasites are thought to control protein activity at the post-translational level, e.g. by protein phosphorylation. In the pathogenic amastigote, the mammalian stage of *Leishmania* parasites, heat shock proteins show increased phosphorylation, indicating a role in stage-specific signal transduction. Here we investigate the impact of phosphosites in the *L*. *donovani* heat shock protein 90. Using a chemical knock-down/genetic complementation approach, we mutated 11 confirmed or presumed phosphorylation sites and assessed the impact on overall fitness, morphology and *in vitro* infectivity. Most phosphosite mutations affected the growth and morphology of promastigotes *in vitro*, but with one exception, none of the phosphorylation site mutants had a selective impact on the *in vitro* infection of macrophages. Surprisingly, aspartate replacements mimicking the negative charge of phosphorylated serines or threonines had mostly negative impacts on viability and infectivity. HSP90 is a substrate for casein kinase 1.2-catalysed phosphorylation *in vitro*. While several putative phosphosite mutations abrogated casein kinase 1.2 activity on HSP90, only Ser_289_ could be identified as casein kinase target by mass spectrometry. In summary, our data show HSP90 as a downstream client of phosphorylation-mediated signalling in an organism that depends on post-transcriptional gene regulation.

## Introduction

*Leishmania donovani* is the causative agent of visceral leishmaniasis, also known as *Kala-Azar*, a generalised infection that causes persistent fever, weight loss, anaemia, and hepato-spleno-megaly in afflicted humans and has a fatal outcome in untreated and treatment failure cases. The annual global incidence is estimated as between 300,000 and 400,000 cases with up to 30,000 fatalities^1^.

The *L*. *donovani* life cycle is bipartite, with a flagellated, elongated promastigote stage that proliferates in the digestive tract of the transmitting sand flies, and an ovoid, aflagellated intracellular stage, the amastigote, which proliferates inside mammalian phagocytic cells. The conversion between the life cycle stages is crucial for parasite survival in sand flies and mammalian hosts and triggered by changes in the environment, i.e. temperature and pH. In keeping with this, *L*. *donovani* promastigotes can be triggered into amastigote conversion by elevated culture temperatures combined with acidic growth medium^2^.

An elevated cultivation temperature also causes an increased synthesis of several parasite heat shock proteins^3–8^ suggesting their involvement in intracellular survival and life cycle control. Heat shock proteins also play other decisive roles in the intracellular amastigote stage, being a prominent part of the protein payload of immune modulatory secreted vesicles known as exosomes. Nearly all major chaperones, including HSP90, HSP70, CPN60.2 and CPN60.3, STIP1 and HSP100 are released into the host cell cytoplasm, with the latter playing a crucial role in the sorting of exosome content. Lack of chaperones in the exosomes abrogates their immune modulatory potential^9,10^.

Chaperone proteins of the HSP90 family play well known roles in the maturation and activation of a multitude of regulatory proteins, such as transcription factors, protein kinases, hormone receptors and cytoskeletal proteins^11,12^ and can even silence mutations in regulatory proteins^13^.

Consequently, HSP90 is an essential protein in all eukaryotic organisms precluding the viability of HSP90 null mutants in any known system. Moreover, HSP90 is a highly abundant protein species, accounting for up to 3% of a cell’s total protein^3,14^. Therefore, reverse genetics approaches, i.e. creating null mutants, to unravel the role and function of HSP90 are likely to fail. The discovery of the HSP90-specific inhibitor geldanamycin (GA)^15^ allowed the pharmacological inactivation of this protein family and the study of the phenotypical consequences in tumor cell lines^16–19^, but also in parasitic microorganisms such as *Leishmania donovani*, *Trypanosoma cruzi*, and *Plasmodium falciparum*^20–22^. Geldanamycin has a specific affinity for the HSP90 ATP-binding pocket, an affinity it shares with another specific inhibitor, radicicol (RAD)^23,24^. Both inhibitor classes cause a cell cycle arrest in logarithmically growing culture cells and indirectly inhibit the maturation and function of HSP90-dependent client proteins, initially offering great promise as anti-cancer drugs^25^. In *Leishmania donovani*, pharmacological inhibition of HSP90 can induce morphological and biochemical promastigote-to-amastigote differentiation^20,26,27^, mimicking the known environmental triggers, i.e. heat shock and low pH, and indicating a pivotal role for HSP90 in environmental sensing and life cycle control.

HSP90 is usually found as a dimer in large complexes known as foldosomes. In their function they depend on ATP hydrolysis and on a cohort of so-called co-chaperones that assist in the recruitment of client proteins and in the regulation of the HSP90 reaction cycle^28,29^. The HSP90 foldosome can vary in composition depending on the client proteins, but usually includes subunits such as HSP70/40, P23, AHA1 and STIP1 (HOP)^29^. HSP90, HSP70 and STIP1 are also known as substrates of protein kinases^30–32^, affecting function and subcellular localisation of these chaperones.

*Leishmania* spp also possess an almost complete set of co-chaperones, with the notable absence of CDC-37, but including the essential co-chaperones STIP1, SGT^27,29,33–35^ and others with less impact on the viability, such as HIP, P23, and AHA1^36–38^. The HSP90 and HSP70 chaperones, but also co-chaperones such as STIP1 and cyclophilin 40, have been shown to be phosphorylated during amastigote stage conversion^34,39^. Mutagenesis of two phosphorylation sites (P-sites) in *L*. *donovani* STIP1 caused either general lethality or no phenotype at all^34^. Mutation of the single P-site in cyclophilin 40 had no phenotypic consequences^40^.

HSP90 is involved in a variety of diverse cellular processes and its complex functions are highly regulated by post-translational modifications, such as phosphorylation, acetylation, SUMOylation, methylation, ubiquitination and *s*-nitrosylation^31,41^. Phosphorylation of HSP90 does not only regulate its activity directly, but also its interaction with other chaperones, co-chaperones, nucleotides and client proteins^32,42–45^. The kinases responsible for phosphorylation of HSP90 include double-stranded DNA protein kinase, B-raf, Akt, c-Src, protein kinase A, Swe^Wee1^, GSK-3beta, casein kinase 1 and casein kinase 2^31,32^.

Essential protein kinases are discussed as attractive drug targets against cancer but also against infectious diseases, such as leishmaniasis. In *Leishmania*, a variety of kinases have already been identified as potential drug targets, such as mitogen-activated protein kinases (MAPKs), cdc-related kinase 3 (CRK3) and GSK3^46–50^. The members of the *Leishmania* casein kinase 1 (CK1) family have also emerged as potential drug targets^51^. The CK1 family consists of multifunctional Ser/Thr protein kinases, characterised by a highly conserved kinase domain and a specific C-terminal domain responsible for kinase regulation and localisation^52^. CK1 isoform 2 (CK1.2) of *L*. *donovani* was identified as exokinase released via immune-modulatory exosomes into the host cell cytosol, where it may phosphorylate and modulate host cell proteins^9,10,53–55^. This kinase was shown to be essential for intracellular parasite survival.

Thus far, *Leishmania* kinases and phosphatases involved in HSP90 modification are poorly understood. Nevertheless, a recent study demonstrated that the *L*. *donovani* MAPK1, whose *L*. *mexicana* orthologue is involved in parasite viability and drug resistance^56^, interacts with HSP90 and HSP70, affects the expression of HSP90 and HSP70 and phosphorylates both chaperones *in vitro*^57^. Given the co-localisation of HSP90 and other major chaperones with CK1.2 in the exosome export pathway^10^, this kinase may also act as upstream kinase for HSP90 regulation.

Thus far, three phosphorylation sites have been identified in the *L*. *donovani* HSP90, Thr_223_, Ser_526_ and Thr_216_^34,58^, all showing increased modification during promastigote-to-amastigote differentiation. However, an analysis of the impact of phosphorylation on HSP90, a key regulator of the *Leishmania* life cycle^20^, was hindered so far by the high copy numbers of HSP90-coding genes in *Leishmania*^59^ and the essential nature of HSP90. However, the introduction of an inhibitor-resistant sequence variant of *L*. *donovani* HSP90, allowing a conditional mutagenesis of this protein^27^, has paved the way for an analysis of HSP90 phosphorylation sites and their impact on fitness, morphology and infectivity. Also, the ability of CK1.2 to phosphorylate HSP90 *in vitro* was tested on a panel of phosphorylation site mutants, identifying at least one CK1.2-specific site.

## Results

### Identification of HSP90 P-sites and mutagenesis

The P-sites at Thr_223_ and Ser_526_ were identified in *L*. *donovani* HSP90 by phosphoproteome analysis^34^.

**Figure 1:**
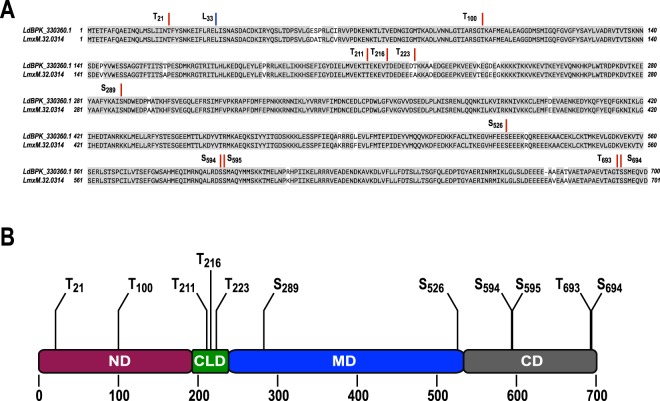
Schematic representation of the targeted phosphorylation sites and domain organisation of HSP90. (**A**) HSP90 sequence alignment for *L*. *donovani* (LdBPK_330360.1) and *L*. *mexicana* (LmxM.32.0314). Phosphorylation sites (see Table 1) are highlighted. (**B**) Putative domain architecture of HSP90 and distribution of phosphorylation sites over N-terminal (ND), charged linker (CLD), middle (MD) and C-terminal domain (CD). Standard one-letter amino acid codes apply.

To identify further phosphorylation sites within the *Leishmania* HSP90, phosphoproteomics analyses were performed on cultured promastigotes, *in vitro* differentiated axenic amastigotes and mouse-lesion-derived amastigotes of *L*. *mexicana*. This led to the identification of seven previously unknown phosphorylation sites at Thr_211_, Thr_216_, Ser_289_, Ser_526_, Ser_594_ and Ser_595_. Fragmentation spectra can be found in the supplementary material (Fig. S1). All sites are conserved in *L*. *donovani* (Fig. 1A). The Thr_223_ P-site of *L*. *donovani* is not conserved in *L*. *mexicana*.

We also identified putative P-sites by literature search and sequence alignments. Two P-sites at Thr_22_ and Thr_101_ are located in the ATP-binding pocket of the *S*. *cerevisiae* HSP90, and Thr to Ile exchanges affect the ATPase activity of ScHSP90^43,60^. In *L*. *donovani* HSP90, these residues are conserved at positions Thr_21_ and Thr_100_ and were mutated identically, namely Thr_21_ to Ile_21_ and Thr_100_ to Ile_100_.

The putative P-sites at Thr_693_ and Ser_694_ were inferred from the known P-sites Thr_725_ and Ser_726_ in the human cytosolic αHSP90 32. A list of the serine and threonine residues targeted for replacement with non-modifiable alanine and/or phosphomimetic aspartate is shown in Table 1 and schematically in Fig. 1.

**Table 1:** List of the Ser and Thr residues in HSP90 that were subjected to mutagenesis. Three-letter amino acid codes apply.

Position	Reference	Constitutive de-phosphorylation	Phosphomimetic exchange	Surrounding Sequence
Thr_21_	(Mollapour *et al*., 2011)	Thr_21_ – Ile_21_	Thr_21_ – Asp_21_	Ser-Leu-Ile-Ile-Asp-**Thr-**Phe-Tyr-Ser-Asn-Lys
Thr_100_	(Hawle *et al*., 2006)	Thr_100_ – Ile_100_	Thr_100_ – Asp_100_	Ile-Ala-Arg-Ser-Gly-**Thr**-Lys-Ala-Phe-Met-Glu
Thr_211_	this paper	Thr_211_ – Ala_211_	Thr_211_ – Asp_211_	Met-Val-Glu-Lys-Thr-**Thr**-Glu-Lys-Glu-Val-Thr
Thr_216_	this paper	Thr_216_ – Ala_216_	Thr_216_ – Asp_216_	Thr-Glu-Lys-Glu-Val-**Thr**-Asp-Glu-Asp-Glu-Glu
Thr_223_	(Morales *et al*., 2010)	Thr_223_ – Ala_223_	Thr_223_ – Asp_223_	Glu-Asp-Glu-Glu-Asp-**Thr**-Lys-Kys-Ala-Ala-Glu
Ser_289_	this paper	Ser_289_ – Ala_289_	Not done	Phe-Tyr-Lys-Ala-Ile-**Ser**-Asn-Asp-Trp-Glu-Asp
Ser_526_	(Morales *et al*., 2010)	Ser_526_ – Ala_526_	Not done	Val-His-Phe-Glu-Glu-**Ser**-Glu-Glu-Glu-Lys-Gln
Ser_594_	this paper	Ser_594_ – Ala_594_	Ser_594_ – Asp_594_	Gln-Ala-Lys-Arg-Asp-**Ser**-Ser-Met-Ala-Gln-Tyr-Met
Ser_595_	this paper	Ser_595_ – Ala_595_	Ser_595_ – Asp_595_	Gln-Ala-Leu-Arg-Asp-Ser-**Ser**-Met-Ala-Gln-Tyr-Met
Thr_693_	(Muller *et al*., 2013)	Thr_693_ – Ala_693_	Thr_693_ – Asp_693_	Glu-Val-Thr-Ala-Gly-**Thr**-Ser-Ser-Met-Glu-Gln-Val-Asp
Ser_694_	(Muller *et al*., 2013)	Ser_694_ – Ala_694_	Ser_694_ – Asp_694_	Glu-Val-Thr-Ala-Gly-Thr-**Ser**-Ser-Met-Glu-Gln-Val-Asp

We have previously established a system for a conditional phenotype analysis of HSP90 mutants *in vitro*. Briefly, a Leu33Ile mutant of HSP90, dubbed HSP90rr, was created which is resistant to the HSP90-specific inhibitor RAD. Ectopic expression of HSP90rr from episomal transgenes has no effect under normal culture conditions, but under RAD challenge, HSP90rr facilitates normal growth, morphology and infectivity. Any mutation introduced into HSP90rr only shows a phenotype under RAD challenge when the endogenously coded HSP90 is inactivated^27^.

Using a non-commercial procedure for targeted mutagenesis^27^ and specific oligonucleotide primer pairs for each target sequence (Table S1), we introduced the desired codon changes into the HSP90rr coding sequence and placed the mutated and verified genes in the shuttle expression plasmid pTLv6^27^. The various mutated HSP90rr transgenes were then stably transfected under G418 selection in *L*. *donovani* strain 1SR. Faithful transcription from the transgenes was verified by transgene-specific RT qPCR (not shown).

The growth kinetics of parasites expressing the transgenes for HSP90wt, HSP90rr and mutants thereof were monitored in the absence of RAD and found to be indistinguishable (not shown), indicating that the expression of HSP90wt from the chromosomal gene copies masks the mutant phenotypes, leading us to conclude that none of the mutations has a dominant negative effect.

### Effect of P-site mutations on *in vitro* growth

To assess the effect of the P-site mutations in HSP90rr on *in vitro* growth, we seeded promastigotes in medium without selective antibiotic and added RAD at 90% growth-inhibitory concentration (IC_90_). Cell density was then monitored over 4 days. The density of HSP90rr cells after 96 h was then defined as 100% growth and parasites expressing the non-RAD-resistant HSP90wt transgene were taken as standard for the loss of HSP90 function.

**Figure 2:**
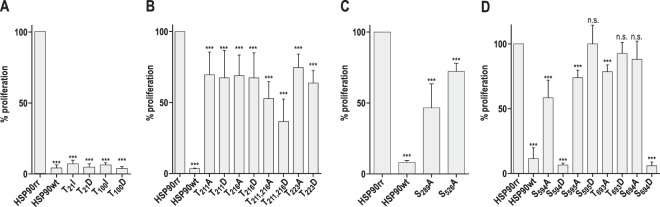
Impact of phosphorylation site mutations on the *in vitro* proliferation. *L*. *donovani* promastigotes were seeded at 1 × 10^6^ cells ml-1 in Medium199 containing radicicol at IC_90_ (batch-dependent; 0,5-1,25 μg/ml) and allowed to proliferate for 96 h. Median (±range) cell density was recorded and plotted as [%] relative to the cell density of promastigotes expressing HSP90rr. Standard one-letter amino acid codes apply. (n.s.): not significant, (***): p ≤ 0.001 by U-test; *n* = 8. (**A**) ATP-binding pocket mutations. (**B**) Charged linker domain mutations. (**C**) Middle domain mutations. (**D**) C-terminal domain mutations.

Mutations in the N-terminal ATPase domain of HSP90rr at Thr_21_ and Thr_100_ both had deleterious effects on the proliferation under RAD inhibition (Fig. 2A). Interestingly, both Thr to Ile (non-phosphorylatable) and Thr to Asp (phosphomimetic exchange) had the same effect, suggesting that the phosphomimetic exchange was not functional.

All three known or suspected P-sites in the charged linker domain (CLD), Thr_211_, Thr_216_ and Thr_223_, were mutated into alanine or aspartate, respectively. All mutations caused a significant ~30% reduction of the *in vitro* growth rates compared to the parental HSP90rr transgene, but no differences between alanine and aspartate exchanges were observed. Combined mutations of both Thr_211_ and Thr_216_ had significant additive effects, reducing growth by up to 65% (Fig. 2B).

Both mutations in the middle domain (MD) of HSP90rr caused a significant growth retardation of 55% for Ser_289_Ala and 25% for Ser_526_Ala (Fig. 2C). Since the MD is thought to specify client protein affinity, phosphorylation-mediated changes may affect client protein recognition.

Two pairs of adjacent P-sites were targeted in the C-terminal domain which is important for the interaction with co-chaperones 27 and for HSP90 dimerisation. For this analysis, the P-sites Ser_594_, Ser_595_, Thr_693_ and Ser_694_ were mutated into Ala or Asp, respectively. While the Ser_594_Ala exchange caused a significant (~50%) reduction of *in vitro* growth, the Ser_594_Asp exchange abolishes parasite growth entirely. The Ser_595_Ala exchange also caused a significant but weaker (~25%) reduction of the *in vitro* growth, but the Ser_595_Asp exchange facilitates parasite proliferation at ~100%. The Thr_693_Ala but not the Thr_693_Asp mutation reduced growth by ~20%. A strong effect was observed for Ser_694_Asp where a permanent negative charge completely abrogates promastigote growth under RAD challenge. By contrast, the Ser_694_Ala exchange had no significant effect (Fig. 2D).

### Effect of phosphosite mutations on promastigote morphology

**Figure 3:**
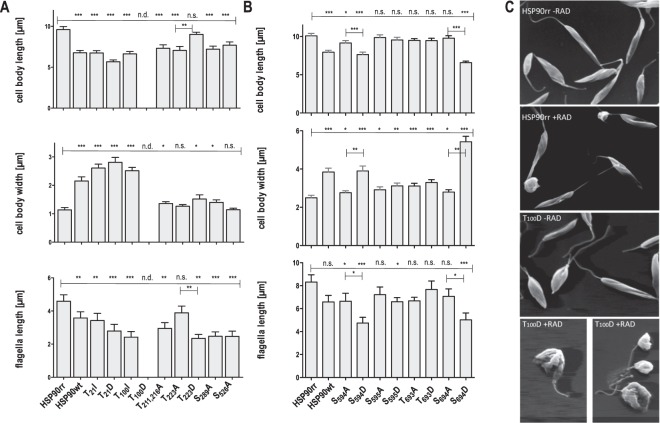
Morphological analysis of recombinant *L*. *donovani* populations. *L*. *donovani* promastigotes were seeded at 1 × 10^6^ cells ml-1 in Medium199 containing radicicol at IC_90_ (batch-dependent; 0,5-1,25 μg/ml) and allowed to proliferate for 72 h. The cells were fixed and then visualised by SEM or fluorescence microscopy using anti-α-tubulin mAb. By using image analysis, the cell body lengths of 50 (in µm, upper panels), the cell body widths of 25 (in µm, middle panels) and the flagella lengths (in µm, lower panels) of 25 randomly selected cells were analysed and mean values were compiled. Bars represent the mean measurements with SEM, n = 25. (n.d.): not detectable, (n.s.): not significant, *p ≤ 0.05, **p ≤ 0.01, ***p ≤ 0.001, respectively, by U-test. (**A**) analysis performed by using SEM images. (**B**) analysis performed by using fluorescence microscopy images. (**C**) SEM images of HSP90rr and HSP90rr-T100D expressing cells -RAD and + RAD.

HSP90 plays a pivotal role in *L*. *donovani* life cycle control. Inhibition of HSP90 by geldanamycin or radicicol induces promastigote-to-amastigote conversion in the absence of elevated temperature and/or acidic pH^20,26,27,61^. Morphologically, this leads to a change of the cell shape from spindle-shaped to ovoid and to the shortening of the flagellum^27^. We therefore analysed length and width of the cell body and length of the flagellum of *L*. *donovani* transfected with the P-site-mutated HSP90rr variants 72 h after RAD treatment, using either scanning electron microscopy (SEM, Fig. 3A)^27^ or anti-tubulin labelling and fluorescence microscopy (Fig. 3B)^38^. The lengths of the mutants were compared to the lengths of HSP90rr and HSP90wt over expressing parasites (Fig. 3A,B).

While the parasites bearing the HSP90rr transgene maintain a spindle shape with a long flagellum (~9 µm cell body length, ~1 µm cell body width, ~4 µm flagella length) after a 72 h RAD treatment, the cells over expressing the non-RAD-resistant HSP90wt are shorter, with a slightly but significantly shorter flagellum (~6 µm cell body length, ~2 µm cell body width, ~3.5 µm flagellar length) (Fig. 3A).

Changing the two N-terminal P-sites Thr_21_ and Thr_100_ to Ile or to Asp abrogates the ability of HSP90rr to promote the promastigote shape under RAD challenge. For all these mutants, we observed a significant reduction of the cell body length to ~5–6 µm, an ovoid cell shape with an increase of the cell body width to ~3 µm and a significant reduction of the flagellum to ~2 µm. The Thr_100_Asp exchange in particular resulted in damaged cells under RAD treatment (Fig. 3C) and non-viable promastigotes. Therefore, it was impossible to obtain measurements of this mutant.

Due to the closeness of the positions Thr_211_ and Thr_216_ we created double Ala-mutants (Thr_211_/Thr_216_Ala) and analysed only the morphology of the double mutant due to its indistinguishable growth phenotype from the single mutants (Fig. 2A). The simultaneous Ala-exchange of these two Thr-residues (Thr_211_/Thr_216_Asp) in the CLD reduces the cell body length significantly to ~6 µm and the flagellum length to ~2.5 µm under RAD. The cell body width is also significantly increased.

Peculiar morphologies correlate with the mutations at Thr_223_. Thr_223_Ala causes a shortening of the cell body (~6 µm), but normal cell body width and flagellar length (~4 µm), reminiscent of metacyclic promastigotes^62^. The cell body length phenotype is reversed in the phosphomimetic Thr_223_Asp mutant (8–9 µm), which however shows a shortened flagellum. This result hints at an involvement of Thr_223_ phosphorylation in the maintenance of the procyclic promastigote.

The middle domain P-sites Ser_289_ and Ser_526_ are also critical for maintaining spindle shape. The Ala-exchanges at these positions render the cells shorter (Ser_289_Ala: ~6 µm, Ser_526_Ala: ~7 µm) with a reduced flagellum (Ser_289_Ala: ~2 µm, Ser_526_Ala: ~2 µm).

The impact of the mutations at the C-terminus of HSP90rr were analysed by fluorescence microscopy using anti-tubulin antibodies. This showed identical effects when comparing HSP90wt and HSP90rr over expressing cells (Fig. 3B). The Ser_594_Ala mutation slightly reduces cell body length (~8 µm) and the length of the flagellum (~5.5 µm). The cell body width is slightly increased (~3 µm). The mutation Ser_594_Asp in contrast has a strong and significant impact on the promastigotes morphology, with a shortening of the cell body length to ~7 µm and the flagellum to ~4 µm. The cell body width of these mutants is significantly enlarged to ~4 µm. The mutations Ser_595_Ala and Ser_595_Asp have no significant effects on the cell body lengths, but increased the cell body widths slightly and decreased the length of the flagella to ~6 µm. Also for Thr_693_Ala and Thr_693_Asp we observed an intermediate impact of the mutations on the cell morphology. Ser_694_Ala has no impact on the cell body length (~10 µm) with a slight increase of the cell body width. The length of the flagellum is slightly but not significantly reduced. These morphological promastigote-like features are reversed by the phosphomimetic exchange Ser_694_Asp. The mutation Ser_694_Asp reduces the cell body length (~7 µm) and the length of the flagellum (5 µm). The cell body width is significantly enlarged to ~5 µm.

The observed impact on the morphologies of the HSP90 variant-expressing parasites largely reflects their growth rates under RAD. One exemption to this correlation is the Thr_223_ site where lack of phosphorylation appears to drive the mutant towards metacyclogenesis.

### Effect of phosphosite mutations on *in vitro* infectivity

**Figure 4:**
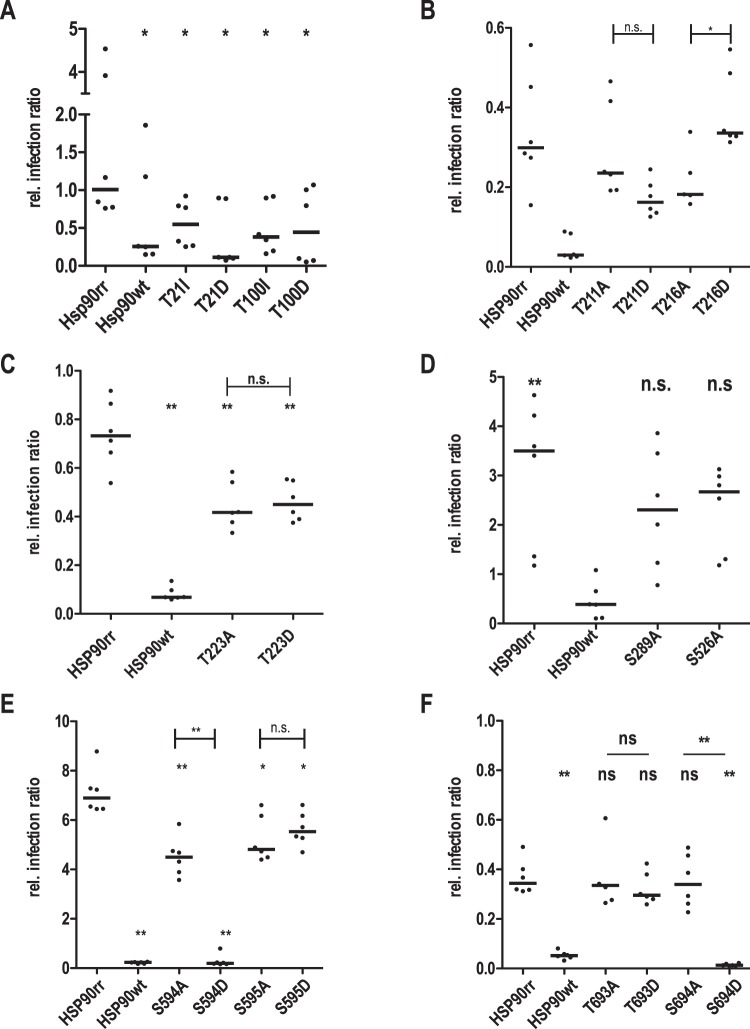
Effects of HSP90 phosphorylation site mutations on the *in vitro* infectivity (**A**–**F**). Recombinant parasites were first pre-treated with RAD for 48 h at IC_90_ and then used to infect bone marrow-derived macrophages (MOI 10:1). After 4 h, free parasites were removed and the infected macrophage cultures were further incubated at 37 °C and 5% CO_2_ for 44 h. Genomic DNAs were isolated from free and attached macrophages and subjected to a duplex qPCR against mouse and *Leishmania* actin DNA (Bifeld *et al*., 2016). The median parasite:host DNA ratio was then compiled. Abbreviations: (n.s.): not significant, *p ≤ 0.05, **p ≤ 0.01 in a one-tailed, paired Student’s t test (**A**), or in a two-tailed U-test (**B**–**F**); *n* = 6.

Several P-sites in HSP90 were identified previously^34^ or in this paper as being preferentially phosphorylated in amastigotes by comparative phosphoproteomics of promastigote and axenic amastigote lysates (see Results, 1st section). While axenic amastigotes are convenient models for stage conversion, we were interested in the impact of phosphorylation on the intracellular survival of true *L*. *donovani* amastigotes. Testing the sensitivity of primary mouse bone marrow-derived macrophages against RAD, we found that the concentrations required to render *L*. *donovani* dependent on the episomal HSP90rr gene variants already affected the ability of the macrophages to support a *Leishmania* infection (not shown). We therefore decided to pre-treat the parasites bearing the HSP90rr transgenes with RAD for 48 h prior to macrophage infection to avoid exposure of the host cells to RAD, as described previously^27^. The effects of the various HSP90rr mutants on the *in vitro* infectivity were analysed 48 h post-infection and compared with the recombinant cells bearing the resistant HSP90rr transgene and the HSP90wt over expressing cells (Fig. 4). We applied a duplex qPCR approach to quantify the ratio of parasite actin to macrophage actin DNA as described previously^63^.

Under these conditions, over expression of HSP90rr prevents a loss of infectivity due to the RAD treatment, resulting in a significantly higher median infection ratio compared with HSP90wt over expressing parasites, with differences slightly varying between individual experiments (Fig. 4A–F).

The Thr_21_Ile mutation resulted in ~50% reduced parasite load, albeit twice as high as the HSP90wt control, while the phosphomimetic exchange Thr_21_Asp reduced the parasite load even below HSP90wt levels. The mutations Thr_100_Ile and Thr_100_Asp both lower the parasite load by ~60% (Fig. 4A). Obviously, the putative P-sites Thr_21_ and Thr_100_ that are located in the ATP-binding domain are not only critical for promastigote growth but also for infection of macrophages. Surprisingly, the Thr_100_ mutants support increased intracellular persistence compared with the HSP90rr control, in spite of their inability to support growth of the promastigote (Fig. 2).

The Ala-exchanges at the P-sites in the charged linker region, Thr_211_Ala and Thr_216_Ala, resulted in non-significantly reduced parasite loads (Fig. 4B). The phosphomimetic exchange Thr_211_Asp further lowered the parasite to host cell ratio, arguing against a simple ionic effect of phosphorylated Thr_211_. Thr_216_Asp by contrast restored infectivity significantly over the Thr_216_Ala mutant (Fig. 4B). Phosphorylation of Thr_216_ therefore appears to boost intracellular persistence of *L*. *donovani*.

The Thr_223_Ala exchange also affected infectivity, reducing parasite loads in macrophages to 57% (Fig. 4C, Table 2). However, the phosphomimetic Thr_223_Asp exchange cannot restore infectivity significantly, also arguing against simple ionic effects of phosphorylated Thr_223_.

**Table 2:** Compilation of results. Relative (in % of HSP90rr values) *in vitro* growth, morphology, infectivity and substrate potential for CK1.2 of the tested mutants.

domains	AA	to	growth [%]	length [%]	width [%]	flagellum [%]	infectivity[%]	CK1.2 Substrate
	**HSP90rr**		100.0	100	100	100	100	*n*.*d*.
	**HSP90**		7.4	71	189	78	25	**+**
N domain	**Thr**_**21**_	I	7.3	70	229	75	29	**±**
		D	5.1	59	247	61	11	*n*.*d*.
	**Thr**_**100**_	I	6.3	69	221	53	38	**±**
		D	3.9	*n*.*d*.	*n*.*d*	*nd*	10	*n*.*d*.
CL domain	**Thr**_**211**_	A	72.9	*n*.*d*.	*n*.*d*	*nd*	79	*n*.*d*.
		D	69.2	*n*.*d*.	*n*.*d*	*nd*	54	*n*.*d*.
	**Thr**_**216**_	A	69.6	*n*.*d*.	*n*.*d*	*nd*	61	*n*.*d*.
		D	69.4	*n*.*d*.	*n*.*d*	*nd*	114	*n*.*d*.
	**Thr**_**211**, **216**_	A	52.9	76	120	64		**−**
	**Thr**_**211**, **216**_	D	37.4	*n*.*d*.	*n*.*d*.	*n*.*d*.	n.d.	*n*.*d*.
	**Thr**_**223**_	A	76.0	74	111	85	57	**−**
		D	62.0	94	134	51	61	*n*.*d*.
M domain	**Ser**_**289**_	A	47.5	75	123	54	66	**+**
	**Ser**_**526**_	A	71.6	80	100	54	76	**−**
C domain	**Ser**_**594**_	A	58.1	91	111	80	65	*n*.*d*.
		D	6.3	76	156	57	3	*n*.*d*.
	**Ser**_**595**_	A	74.0	98	117	87	70	*n*.*d*.
		D	98.6	95	125	79	80	*n*.*d*.
	**Ser**_**594/5**_	A		*n*.*d*.	*n*.*d*.	*n*.*d*.	*n*.*d*.	**−**
		D		*n*.*d*.	*n*.*d*.	*n*.*d*.	*n*.*d*.	*n*.*d*.
	**Thr**_**693**_	A	77.9	94	125	80	91	**±**
		D	93.1	94	132	92	81	*n*.*d*.
	**Ser**_**694**_	A	88.2	97	112	85	93	**±**
		D	6.5	65	217	60	4	*n*.*d*.

The phenotypes of Ser_289_Ala and Ser_526_Ala mutations, which are located in the middle domain, are not significant and suggest a rather limited effect on virulence (Fig. 4D).

The Ser_594_Ala, Ser_595_Ala and Ser_595_Asp mutations in the C-terminal region of HSP90rr reduce infectivity slightly but significantly by ~20–30% (Fig. 4E). By contrast, the cells expressing the phosphomimetic Ser_594_Asp mutant were unable to establish an infection (Fig. 4E). This is presumably caused by the inability of these transgenic parasites to grow under RAD *in vitro* (Fig. 2D).

We also tested the effects of the mutated putative P-sites Thr_693_ and Ser_694_ located directly in front of the STIP-1 recognition motif 27 at the extreme C-terminus of HSP90. The exchanges Thr_693_Ala, Thr_693_Asp and Ser_694_Ala do not affect infection ratios. However, the phosphomimetic Ser_694_Asp exchange abrogates intracellular survival completely (Fig. 4F). Like Ser_594_Asp, the Ser_694_Asp mutant of HSP90rr is unable to promote promastigote proliferation under RAD (Fig. 2D), suggesting an inability to tolerate the pre-treatment before infection.

### Identification of HSP90 as *in vitro* target of casein kinase1.2

To gain insight into the mechanisms involved in HSP90 regulation, we investigated whether kinases that have been described to target mammalian HSP90 may also target *Leishmania* HSP90. In particular, we focused on three protein kinases, GSK-3, CK1.2 and DYRK1. Of these, GSK-3 and CK1.2 have been shown to phosphorylate the C-terminal domain of mammalian HSP90. This phosphorylation is particularly important to prevent the binding of HSP90 to CHIP, a co-chaperone containing an ubiquitin ligase activity and thus to prevent the client protein from being degraded^32^. We also selected DYRK1 as it has been shown to be a priming kinase for GSK-3; it phosphorylates the substrates on a specific site which can be recognised and phosphorylated by GSK-3^64^. Therefore, we expressed and purified recombinant *L*. *major* CK1.2 51, *L*. *major* GSK-3^65^ and *L*. *infantum* DYRK1 to perform *in vitro* kinase assays using recombinant *L*. *donovani* HSP90wt.

**Figure 5:**
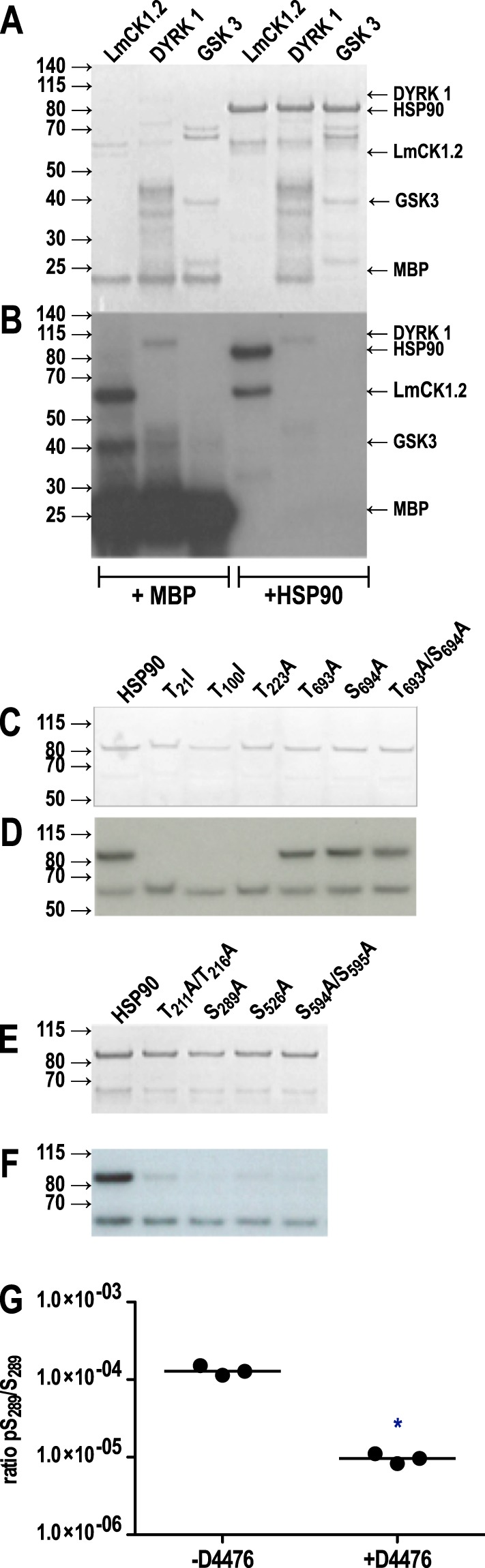
Protein kinase of HSP90. (**A**) Three protein kinases, rLmCK1.2, rDYRK 1, and rGSK 1, were incubated with γ-^32^P-ATP with MBP or with HSP90wt as substrate as indicated below and analysed by SDS-PAGE and Coomassie Brilliant Blue staining. The positions of protein size markers are indicated to the left, the positions of the kinases and of HSP90 are indicated on the right. (**B**) As in (**A**), but autoradiograph. (**C**,**D**) *In vitro* protein kinase assay of rLmCK1.2 with HSP90 and 5 phosphosite mutants thereof, using γ-^32^P-ATP. (**C**) shows a Coomassie Brilliant Blue stain, (**D**) shows the corresponding autoradiograph. (**E**,**F**) As in (**C**,**D**), but with 4 other phosphosite mutants of HSP90. (**G**) Mass spectrometry-based quantification of the phosphoserine/serine ratio at Ser_289_ without (−D4476) or with (+D4476) a CK1.2-specific inhibitor. The y-axis is in log_10_ scale. All greyscale images were cropped from contiguous parts of the original image. Digital enhancements, using Adobe Photoshop CS3, were performed over the entire greyscale images and restricted to tonality optimisation and size adjustments.

As shown in Fig. 5B lane 4, we detected a signal at 80 kDa revealing the transfer of ^32^P onto HSP90 by CK1.2 but not by GSK-3 or DYRK1 (lanes 5 and 6). In contrast, all three kinases were able to phosphorylate myelin basic protein (MBP), a canonical kinase substrate (lanes 1–3). Since we can exclude MBP autophosphorylation^51^, these data suggest that HSP90 is a substrate of CK1.2, which is consistent with previous findings on mammalian HSP90, but not a substrate of GSK-3 or DYRK1. This is different from mammalian HSP90 where it has been shown that mammalian GSK-3 phosphorylates HSP90 at its C-terminus^32^. This may be due to the use of the *L*. *major* (GSK3 and CK1.2) and *L*. *infantum* (DYRK1) orthologues in our *in vitro* assay; however, the sequence identity between the GSK3 of *L*. *major* and *L*. *donovani* is 98%, and the sequence identity between the DYRK1 of *L*. *infantum* and *L*. *donovani* is 99%, making a complete change of client specificity very unlikely.

### Identification of the P-site(s) targeted by CK1.2

Among the P-sites that we identified, the residues Thr_211_, Thr_216_, Thr_223_, Thr_693_ and Ser_694_, match the canonical consensus site for CK1 family members: S/T(X)_2–3_S/T or D/E(X)_2–3_S/T^66^. To identify which of the previously studied P-sites may be targeted by CK1.2, we expressed HSP90 with serine or threonine to alanine mutations to test which mutation abrogates HSP90 phosphorylation by CK1.2. We tested mutants for all known or suspected P-sites since members of the CK1 family can phosphorylate canonical as well as non-canonical sites such as SLS-Xn-(E/D)n^67^ or K/R-X-K/R-(X)_2_-S/T^68,69^. We performed *in vitro* kinase assays using recombinant CK1.2 with HSP90wt or HSP90 phosphosite mutants as substrates. As shown in Fig. 5D, HSP90-Thr_693_Ala, -Ser_694_Ala and -Thr_693_Ala/Ser_694_Ala mutants are still phosphorylated by CK1.2 at levels comparable with the wild type control, although we observe that the levels of Ser_693_Ala and Ser_693_Ala/Thr_694_Ala are slightly lower than those of Thr_694_Ala. By contrast, phosphorylation by CK1.2 was abrogated in Thr_21_Ala, Thr_100_Ala, Thr_211/216_Ala, Thr_223_Ala, Thr_289_Ala, Ser_526_Ala and Ser_594_Ala and Ser_595_Ala mutants of HSP90 (Fig. 5D,F).

We also performed a “cold”, i.e. non-radioactive *in vitro* kinase assay with recombinant HSP90wt and CK1.2 in the presence or absence of 10 µM D4476, a specific CK1 inhibitor^51^. The samples of three independent kinase assays were analysed by mass spectrometry to identify the sites phosphorylated by CK1.2. We found only one site, Ser_289_ (Fig. 5G), which is phosphorylated in the presence of CK1.2 but not in the presence of CK1.2 + D4476, suggesting that CK1.2 targets Ser_289_. No phosphorylation was detected at Thr_211_, Thr_216_, Ser_526_, Ser_594_, Ser_595_ and Thr_223_ in our dataset (data not shown), suggesting that they are not targeted by CK1.2. We cannot exclude that the sites Thr_21_, Thr_100_, Thr_693_ and Ser_694_ may be true CK1.2 sites as the peptides containing those sites were not detected in our mass spectrometric analyses. However, those sites are only presumed phosphorylation sites based on analogies with model HSP90s. Moreover, only Thr_693_ and Ser_694_ resemble canonical CK1 sites.

In summary (Table 2), mutations at the established and putative phosphorylation sites in the *L*. *donovani* HSP90 result in a variety of phenotypic manifestations. The most drastic and general effects are observed after introducing mutations at sites within the highly conserved ATP-binding domain. These HSP90rr variants apparently cannot support growth or infectivity under RAD challenge. Mutation at sites in the charged linker domain show moderate effects on growth and infectivity. Middle domain site mutations result in moderate to strong growth inhibition, morphologic changes and a moderate loss of infectivity, even though one of them is established as the only CK1.2 phosphorylation site. In the C-terminal domain we observe the strongest effects for two of the phosphomimetic Ser-Asp exchanges with no or moderate impact of Ser/Thr to Ala mutations. Clearly, permanent negative charges at these sites are not conducive to parasite fitness. At other sites, Ser/Thr to Asp restore growth, morphology and/or infectivity to a various extent compared with the Ser/Thr to Ala mutations.

## Discussion

Trypanosomatida and perhaps the entire phylum Euglenozoa lack inducible transcription as a means for gene expression control^70,71^. The existing genome databases contain no confirmed genes for transcriptional regulators and cis-regulatory DNA sequence elements such as gene promoters and enhancer elements known from crown group Eukaryota. Moreover, the established mode of multicistronic transcription precludes individual transcriptional control of gene expression^71,72^. Cis-splicing is very rare but trans-splicing has been discussed as point of regulation^73^. This leaves mRNA processing and stability, translation, protein folding and post-translational protein modifications as conceivable targets of gene expression control.

The major chaperone protein HSP90 is subject to various post-translational modifications such as phosphorylation, acetylation, *s*-nitrosylation, oxidation, SUMOylation or ubiquitination^31,74^. Post-translational modifications of HSP90 are known to be involved in the fine-tuning of the chaperone cycle which includes conformational changes, ATPase activity, interaction with co-chaperones and the recruitment and activation of various client proteins^31,32,42,75^. Differences in this fine-tuning processes also adapt HSP90 regulation and function to the unique intracellular environments of different organisms or to various physiological states within different tissues of metazoans^29,76^.

Previous work showed that HSP90, HSP70 and the co-chaperone STIP1 are subject to increased phosphorylation during the promastigote-to-amastigote differentiation of *L*. *donovani*^34^. These data suggested that the function of HSP90 is adaptable to the different environments of the distinct life cycle stages. It was previously described that a heat shock of yeast cells decreases the phosphorylation level of HSP90, while an opposite effect was observed looking at the turnover of phosphate groups in heat-shocked HeLa cells^77,78^. Phosphorylation of this essential chaperone is one possible mechanism to adapt its function to various environmental conditions and in a species-specific manner.

This paper focuses on the impact of HSP90 phosphorylation by introducing amino acid exchange mutations into the radicicol-resistant HSP90rr variant^27^ and monitoring their impact on *in vitro* promastigote proliferation, morphology, *in vitro* infectivity and casein kinase 1.2 substrate properties.

Phosphorylation sites are distributed over the whole polypeptide sequence of HSP90 and all putative domains. In our study, we included sites shown as phosphorylation sites for *L*. *mexicana* HSP90 (Thr_211_, Thr_216_, Ser_289_, Ser_594_, Ser_595_), *L*. *donovani* HSP90 (Thr_223_, Ser_526_) and putative sites identified by analogy (Thr_21_, Thr_100_, Thr_693_, Ser_694_).

We observed that amino acid exchanges at the two putative phosphorylation sites Thr_21_ and Thr_100_ which are implicated in the activity of the ATPase domain from previous work in *Saccharomyces cerevisae*^44,60,79^, severely inhibit growth of the promastigote stage. Even phosphomimetic exchanges to Asp in both positions cannot restore the proliferation capacity. We can offer three explanations for this observation, (i) the exchanges may render HSP90 non-functional as chaperone, (ii) the structural changes caused by the amino acid exchanges in the nucleotide binding pocket may impair ATP turnover, or (iii) the changes may render HSP90rr sensitive to RAD again. The latter view is supported by the finding that expression of these four mutant HSP90rr variants results in phenotypes indistinguishable from the cells that are over expressing the RAD sensitive HSP90wt (Table 2). While phosphorylation by casein kinase 2 at the residue Thr_22_, equivalent to Thr_21_ in *Leishmania* HSP90, was established for the yeast HSP90^43^, it was also shown that Thr_22_Ala and Thr_22_Glu amino acid exchanges impact on ATPase activity and interaction of HSP90 with co-chaperones such as AHA1 and increase the sensitivity against RAD and GA^44^. However, we could recently demonstrate that lack of AHA1 in *Leishmania* has no impact on RAD sensitivity^38^, arguing against an impaired HSP90-AHA1 interaction as the cause for the observed, severe phenotypes. Testing the mutants *in vitro* for ATPase activity, RAD sensitivity and AHA1 binding may shed light on this phenomenon in the future.

Ala and Asp exchanges at Thr_211_ and Thr_216_ in the highly diverged charged linker domain have opposing effects on the parasites’ infectivity. A stable negative charge at position 211 reduces parasite loads by almost 50%, while the effect of a non-phosphorylatable Ala at 216 is reverted by the phosphomimetic Asp exchange. Although neither site could be identified as CK1.2 phosphorylation site, a double Thr-Ala exchange at 211 and 216 abrogates CK1.2 phosphorylation of HSP90 and causes a reduction of the flagellum. Another site, Thr_223_, also in the CL domain and specific for Trypanosomatida HSP90s, moderately affects growth and infectivity, as well as CK1.2-dependent phosphorylation, but is no CK1.2 site itself. Of note are the effects on the morphology where abrogation of phophorylation (Thr to Ala) reduces cell body length, but not flagellar length, thereby resembling the shift from procyclic to metacyclic promastigotes^80^. This is a first hint at a possible role of HSP90 in the transition from proliferative procyclic promastigotes to the infective metacyclic form. The Thr_223_Asp exchange restores cell body length, but increases diameter and reduces flagellar length, resembling early amastigote development. This fits with the identification of Thr_223_ as an amastigote-specific phosphorylation site^34^.

In the middle domain, which in model HSPs has a function in client protein recognition^60,81^, we analysed two sites, Ser_289_ and Ser_526_. Ser_289_ has an impact on proliferation and the maintenance of promastigote morphology. It was also identified as the only CK1.2 site known so far in HSP90, and its replacement with Ala reduces *in vitro* infectivity by 24%. Ser_526_, an amastigote-specific site^34^ moderately affects growth, virulence, flagellar length and CK1.2 phosphorylation, either at Ser_289_ or at Ser_526_, since this site was not detected in mass spectrometry. It is conceivable that the effects of Thr_211/216_, Thr_223_ and Ser_526_ are at least in part due to their impact on Ser_289_ phosphorylation by CK1.2. The question remains open how mutations at Thr_21_, Thr_100_, Thr_211,216_, Thr_223_,but also Ser_526_, Ser_594_, and Ser_595_ can affect phosphorylation at Ser_289_.

The C-terminal domain of HSP90 chaperones is known to facilitate dimerisation and interaction with co-chaperones STIP1^27^ and prolyl-peptidyl isomerases such as cyclophilin 40^40,82^ and others. Four phosphorylation sites were detected or inferred by analogy. Replacement of Ser_594_ caused a ~40% reduction of *in vitro* growth and infectivity while the Ser_694_ to Ala change had little impact. However, the phosphomimetic Ser-to-Asp exchange in these positions completely abrogated HSP90-dependent growth and infectivity. We conclude that permanent negative charges in these positions have a deleterious effect on the HSP90 functionality. The HSP90 reaction cycle is known to include the ordered assembly and disassembly of HSP90-co-chaperone complexes at the C-terminal domain^83^, possibly requiring frequent reversal of post-translational modifications, such as phosphorylation. A permanent negative charge may therefore impede the ordered transitions of the HSP90 foldosome complex. The effects observed for mutation in two other sites, Ser_595_ and Thr_693_, are moderate by comparison.

One lesson from our data is the strong deleterious effects of presumed phosphomimetic Asp mutations. Unlike MAP kinases where Asp replacements in the activation sites render downstream kinases permanently active, the situation in HSP90 with its multiplicity of active phosphorylation sites and a complex reaction cycle with multiple partnering chaperones and co-chaperones may be too complicated for simplistic approaches. Another problem resides in our experimental set-up where we express RAD-resistant, mutated HSP90rr before a strong background of RAD-sensitive, endogenous HSP90. A treatment with RAD is necessary to express the mutant phenotypes, yet one cannot exclude interactions and even heterologous complexes of HSP90rr and HSP90 monomers. For example, the HSP90-STIP1 interaction is not affected by RAD treatment^27^, opening up the possibility that phenotypes of mutations outside the ATP binding pocket are “watered down” by interaction with endogenously coded HSP90wt. To rectify this problem, we hope for a future implementation of advanced reverse genetics such as CRISPR/cas9 based gene editing^84,85^ to eliminate the entire cluster of ~17 tandemly arranged HSP90 gene copies and to express the mutant HSP90s before a null mutant background.

So far, not much is known about the upstream and downstream players in the presumed signal transduction pathways. In the eukaryotic model organisms, from yeasts to humans, signal transduction pathways govern the activity of many gene expression regulators through reversible phosphorylation^86^. Many of these regulators are trans-acting transcription factors, a protein class that is almost entirely absent from the *Leishmania* proteome^71^. Therefore, post-transcriptional regulation, in particular on the protein synthesis and protein modification levels, are important for Trypanosomatida^49,87^. How these processes are linked to the signal transduction pathways remains one of the most important questions in the field. Since HSP90 and its co-chaperones are integral parts of the signal transduction pathways governing the *Leishmania* life cycle^20,27,61^ and are targets of stage-specific phosphorylation events^34^, the post-translational modification of HSP90 is likely to have multiple effects on *Leishmania* viability and fitness, some of which were uncovered in our analysis.

## Materials and Methods

### *L*. *donovani* culture, genetic complementation and *in vitro* infection model

*L*. *donovani* 1SR^88^ were cultivated as described^27^. Batch-specific IC_90_ were determined for radicicol (Sigma-Aldrich, München, Germany) prior to each experiment. Concentration range was 0.5–1.25 μg/ml. Electrotransfection and antibiotic selection of *L*. *donovani* promastigotes was carried out as described^27^.

### *In vitro* infection model

*In vitro* infection of murine bone marrow-derived macrophages with RAD-treated *L*. *donovani* promastigotes was performed as described earlier^27^ with two modifications: (i) bone marrow macrophage precursor cells were differentiated using 10–30% supernatant of LADMAC cells^89^, and (ii) quantification of infections were performed by quantitative PCR^63^. Adherent BMMs were infected at a multiplicity of infection of 10 parasites per macrophage. After 4 hours of incubation at 37 °C in DMEM/F-12, supplemented with GlutaMAX, free parasites were removed by 3 washing steps with PBS, and the infected cells were incubated at 37 °C and 9% CO_2_ for another 44 hours. At 48 h post-infection, free cells in the culture supernatant and attached cells were pooled by sedimentation and lysed. Genomic DNA (gDNA) was isolated from the infected cells using the ISOLATE II Genomic DNA Kit (Bioline, Luckenwalde, Germany). Parasites were then quantified by semi-quantitative real-time PCR (qPCR) targeting host cell and parasite actin-coding genes with double labeled probes and using total parasite and host gDNA as the template^63^. The relative parasite load was defined as the ratio of parasite actin DNA against mouse actin DNA.

### Targeted mutagenesis of HSP90-coding sequences

The generation of the RAD-resistant HSP90 variant (HSP90rr) was described earlier^27^. Plasmid pUC:HSP90rr served as template for site-directed mutagenesis. Primers (Supplementary data, Table S1) were phosphorylated using ATP and polynucleotide kinase (PNK, New England Biolabs)^90^ and then used to prime a PCR amplification of pUC:HSP90rr using the iProof-PCR kit for GC-rich DNA (#172–5320) from Bio-Rad Laboratories (München, Germany). Following amplification (30 cycles), the linear PCR product was subjected to ligation (3 h, RT) to form circular plasmids. These were then used to transform competent *E*. *coli* DH5-α cells (#18265–017, Invitrogen, Karlsruhe, Germany). After amplification and purification^90^ via caesium-chloride density gradient ultracentrifugation (50% w/v CsCl, 6 h, 90,000 rpm, 20 °C, rotor NVT90, Beckman, Krefeld, Germany), the pUC:HSP90rr-derived mutants were verified by DNA sequencing (LGC Genomics, Berlin, Germany).

The derivative of the plasmid pJC45^91^, pJC45:HSP90, was also used for site-directed mutagenesis. The same set of primers (Table S1) was used. PCR, ligation, transformation of *E*. *coli* and CsCl purification of plasmids were performed the same as for the site-directed mutagenesis of pUC:HSP90rr.

### Construction and preparation of recombinant DNA

The expression plasmid pTLv6 has been described^27^. Mutated HSP90 coding sequences derived from pUC:HSP90rr were excised with enzymes *Kpn*I and *Bam*HI (compatible with *Bgl*II sticky ends) and ligated into linearised pTLv6 to create the pTLv6:HSP90rr expression plasmids coding for the various HSP90rr variants with P-site codon exchanges.

### Expression profiling

Semi-quantitative real-time RT-PCR was performed essentially as described^92^. HSP90 transgene-specific primers were pTLv6.HSP90.F2 (CGACGAGGAGGAGGAGGCAG) and pTLv6.HSP90.B1 (GCCAGTACATCACAAGACTC ATAGATCC). HSP90 mRNA abundance was calculated relative to the actin signal.

### Scanning electron microscopy (SEM)

*Leishmania* cells were cultivated for 72 h without RAD or with the IC_90_ of RAD. The treated and non-treated cells were then washed twice in PBS, fixed in 2% glutaraldehyde in sodium cacodylate buffer and postfixed with 1% osmium. Samples were dehydrated at increasing ethanol concentrations (30–100%). After critical point drying, samples were treated with gold and analysed on a Philips SEM 500 electron microscope. Images were taken using a conventional 35 mm camera, and the developed black-and-white films were digitalised using a HAMA 35 mm film scanner.

### Fluorescence microscopy

Promastigote cells were incubated for 72 h without RAD or with the IC_90_ of RAD. After the incubation period, cells (1 × 10^7^ cells) were sedimented, washed twice with PBS and suspended in 1 ml of PBS. Aliquots of the suspension (2 × 10^5^ cells) were applied on microscopic slides and air-dried. After fixing the cells for 2 min in ice-cold methanol, the slides were air-dried for 20 min. Non-adherent cells were removed by gentle washing (0.1% Triton X-100 in PBS) followed by incubation in blocking solution (2% BSA, 0.1% Triton-X 100 in PBS). Slides were then incubated for 1 h with monoclonal mouse anti-tubulin (Sigma-Aldrich, München, Germany, 1:4000), washed thrice and then incubated for 1 h with anti-mouse Alexa 594 (Dianova, Hamburg, Germany, 1:250) and DAPI (Sigma-Aldrich, München, Germany, 1:25). After washing the slides thrice, Mowiol and coverslips were applied and the slides were left to dry for 24 h at 4 °C. Fluorescence microscopy was carried out on an EVOS FL Auto epifluorescence microscope (Life Technologies, Darmstadt, Germany).

### Phosphoproteomics

*Leishmania mexicana* MNYC/BZ/62/M379 promastigotes were cultured in SDM-79 medium^93^ supplemented with 10% heat-inactivated FCS (Sigma, Steinheim, Germany), 7.5 µg ml^−1^ hemin (Sigma, Steinheim, Germany) and 100 U ml-1 penicillin/100 µg ml-1 streptomycin (Pen/Strep; Gibco, UK). Cultures were incubated at 27 °C until they reached a density of 4–5 × 10^7^ parasites ml-1 (late-log phase). Cultures were either harvested or differentiated into amastigotes by inoculation in Schneider’s Drosophila medium (PAN Biotech, Aidenbach, Germany) supplemented with 20% FCS (PAN Biotech), 2 mM L-glutamine, 100 U ml-1 Pen/Strep, and 20 mM 2-morpholinoethane sulfonic acid monohydrate [MES, pH 5.5] (Serva, Heidelberg, Germany). Cultures were incubated at 34 °C/5% CO_2_ for 72 h. Parasites were harvested by centrifugation at 2000 × g, 4 °C, and washed in 1) ice-cold wash buffer (21 mM HEPES pH 7.5, 137 mM NaCl, 5 mM KCl) and 2) ice-cold wash buffer with protease and phosphatase inhibitors (1 mM Na-orthovanadate, 0.1 µM okadaic acid, 10 mM NaF, 10 mM *o*-phenanthroline, EDTA-free protease inhibitors). For lysis, parasites were resuspended in a solution of 7 M urea, 2 M thiourea, 40 mM Tris, 1% n-octyl-β-D-glycopyranoside, 1 mM MgCl_2_, 1 mM *o*-phenanthroline, 300 U benzonase, 1 mM Na-pervanadate (Na-orthovanadate activated in 18% H_2_O_2_), protease inhibitors (Roche EDTA-free protease inhibitor tablets), and phosphatase inhibitor cocktails (P2850 and P5726 from Sigma), and sonicated for 3 × 15 s on ice. Lysates were incubated at −80 °C for 30 min prior to reduction (50 mM DTT) and alkylation with 50 mM iodoacetamide. Proteins were precipitated in an 8-fold excess of ice-cold acetone-ethanol, 1:1, v/v, by overnight incubation at −20 °C.

Protein precipitates were reconstituted in 6 M urea/2 M thiourea and diluted in 50 mM NH_4_HCO_3_ for digestion with trypsin at a 1:75 enzyme-substrate ratio overnight. Digestion was quenched by addition of formic acid (FA) to a final concentration of 1%. Aliquots of 330 µg digested proteins were used for phosphopeptide enrichment by TiO_2_ (Titansphere, 5 µm; GL Sciences, Tokyo, Japan). Enrichment was performed with home-made columns packed in p200 pipette tips, essentially as described before^94^. Samples for LC-MS/MS were prepared by desalting on Stop And Go Extraction (STAGE)-tips^95^. A plug of C8 (phosphopeptides) or C18 (non-phosphorylated peptides) Empore disc (3 M Bioanalytical Technologies, St. Paul, MN, USA) was placed in the thin end of an ordinary p200 pipette tip and washed in 100% acetonitrile (MeCN). The reversed-phase (RP) material was equilibrated with 5% FA and the sample loaded in a 1:1 sample-to-5% FA volume ratio. Peptides were eluted into a clean microcentrifuge tube by 70% MeCN/5% FA and vacuum-dried. Upon preparation for LC-MS/MS analysis, peptides were reconstituted in 0.4 µl 100% FA and diluted with a suitable volume of 0.1% FA. Samples were analysed on LTQ Orbitrap XL mass spectrometers (Thermo Fisher Scientific, Bremen, Germany). For peptide separation, an Easy-nLC™-system (Thermo Fisher Scientific (previously Proxeon), Odense, Denmark) was coupled directly to the mass spectrometer. The Easy-nLC™ was fitted with a home-made analytical column (50 µm i.d.; 100 µm o.d., 18 cm) (ReproSil-Pur C18-AQ, 3 µm; Dr. Maisch, GmbH, Ammerbuch-Entringen, Germany). Phosphopeptides were analysed using collision induced dissociation (CID) MultiStage Activation (MSA) in an MS/MS Top10 set-up for fragmentation of the 10 most intense peptide peaks in each MS scan with 45 seconds exclusion time.

### Phosphoproteomics data analysis and phosphorylation validation

The LCMS data were processed with DTASuperCharge^96^ and searched against the TriTrypDB *L*. *mexicana* database (version 4.0) (TriTrypDB, http://tritrypdb.org/tritrypdb) using an in-house Mascot server (version 2.2.06, Matrix Science, London, UK). Mascot searches were conducted with 5 ppm peptide tolerance and 0.6 Da fragment ion tolerance. For database searches, cysteine carbamidomethylation was included as a fixed modification, while oxidation of methionine, Gln → pyro-Glu (N-term Q), Glu → pyro-Glu (N-term E), pyrocarbamidomethyl (N-term C) and phospho (S, T, and Y) were added as variable modifications. Data sets were compiled in Scaffold (version 3.3.1, Proteome Software Inc., Portland, OR, USA) with a protein threshold of minimum 95% and a peptide threshold of minimum 95% for at least 1 peptide. The phosphorylated proteins fulfilling these criteria were further evaluated in Scaffold PTM (version ScaffoldPTM_1.1.3, Proteome Software Inc., Portland, OR, USA).

Phosphorylation sites were validated by a semi-automatic approach, applying Ascore^97^ in combination with Mascot Delta Score^98^ and manual inspection of selected spectra. Phosphorylation sites with Ascores >19 were accepted unconditionally.

### Phosphoproteomic identification of CK1.2 phosphosites in HSP90

For identification of CK1.2 phosphosites, we performed three independent cold kinase assays that were then transferred into fresh tubes to perform in-solution digestion, according to standard protocols. Briefly, after reduction (to a final concentration of 5 mM DTT) and alkylation (to a final concentration of 10 mM iodoacetamide) samples were diluted 10-fold with ammonium bicarbonate and incubated overnight with 0.2 µg trypsin/LysC (Promega) at 37 °C. Samples were then loaded onto a homemade C_18_ stage tip for desalting. Desalted samples were reconstituted in 2% MeCN/0.3% TFA and analysed by nano-LC-MS/MS by using an RSLCnano system (Ultimate 3000, Thermo Scientific) coupled to an Orbitrap Fusion Tribrid mass spectrometer (Thermo Fisher Scientific). Peptides were analysed using HCD fragmentation with normalised collision energy of 30, in a top speed mode with 45 seconds exclusion time. Spectra were interrogated by Sequest^TM^ through Proteome Discoverer^TM^ 2.1 (Thermo Scientific) with the in-house database containing the sequences of the *Leishmania* HSP90, the *Leishmania* casein kinase 1 (E9AHM9, E9AHM8 and A4IAZ8) and the most abundant contaminants (244 protein sequences). Enzyme specificity was set to trypsin and a maximum of two missed cleavage site were allowed. Oxidised methionine, N-terminal acetylation, phosphorylation of Ser, Thr and Tyr and carbamidomethyl cysteine were set as variable modifications. Maximum allowed mass deviation was set to 10 ppm for monoisotopic precursor ions and 0.6 Da for MS/MS peaks. The resulting files were further processed using myProMS^99^. FDR calculation used Percolator and was set to 1% at the peptide level for the whole study. We validated phosphorylated peptides by combining the phosphoRS information and by manually inspecting the peak assignment.

To quantify the phosphorylated S289 peptide, we extracted from the MS survey of the nanoLC-MS/MS raw files the extracted ion chromatogram (XIC) signal by using the retention time and m/z values of the well characterised tryptic peptide ions using the Xcalibur softwares (manually). XIC areas were integrated in Xcalibur under the QualBrowser interface using the ICIS algorithm. Areas were normalised by using the non-phosphorylated ion’s signal^100^. Mean values and standard deviation were calculated from three independent experiments.

### Recombinant expression of the mutated HSP90 variants

The constructs for the expression of recombinant wild type or mutated HSP90 were introduced into *E*. *coli* BL21(DE3) [pAPlacI^Q^] and expression was induced using 0.4 mM IPTG at 37 °C for 2 h^101^. The His-tagged proteins were purified using a Ni-NTA column (Qiagen), as per the manufacturer’s instructions. Purity of the proteins was verified by SDS-PAGE and Coomassie Brilliant Blue staining. The purified proteins were used for the kinase assays.

### Expression and purification of recombinant LmCK1.2-V5-His_6_, GSK3 and DYRK

The *L*. *major* Friedlin GSK-3 short open reading frame (LmjF18.0270, LmaGSK-3s) was amplified from genomic DNA, cloned into the pTriEx-1.1 vector (Novagen) as previously described^48,102^, while a synthetic ORF of the *L*. *infantum* DYRK1 (LinJ.15.0180), optimised for codon usage in *E*. *coli*, was synthesised in pET14b (EMBL, BamHI/XhoI sites) to encode a His_6_-LinDYRK1 protein (gift from Dr Vinicius Pinto Rocha and Dr Milena Soares). LmCK1.2-V5-His6 (LmjF.35.1010)^51^, GSK-3 and DYRK1 were introduced into *E*. *coli* BL21 Rosetta. The expression of recombinant kinases was induced for 4 hr, with 1 mM IPTG at 37 °C or RT for GSK-3 and DYRK1 respectively, while CK1.2 was expressed with 0.02% (w/v) arabinose at RT as previously described^51^. The His-tag proteins were purified using Co-NTA agarose (Pierce) as previously described^51^.

### *In vitro* kinase assay

*In vitro* kinase assays were performed as described^51^. Briefly, recombinant CK1.2 (rCK1.2), GSK-3 and DYRK1 were assayed in buffer C (60 mM β-glycerophosphate, 30 mM p-nitrophenyl phosphate, 25 mM MOPS, 5 mM EGTA, 15 mM MgCl_2_, 1 mM dithiothreitol, 0.1 mM sodium vanadate, pH 7.0) with 15 µM γ-^32^P-ATP in 30 µl final volume. For the identification of the CK1.2 phosphosites in HSP90, a similar experiment was performed using CK1.2, recombinant HSP90 and cold ATP at 15 µM. We used the following substrates: 2 µg of either recombinant HSP90, recombinant HSP90 mutants or 4 µg of myelin basic protein (MBP, Sigma-Aldrich), except if stated otherwise. The MBP used was batch-tested previously for absence of autophosphorylation^51^. After a 30 min incubation at 30 °C in a 20 µl reaction volume, the reaction was stopped by adding 1 volume 2 × loading buffer^103^. The incorporation of γ-^32^P-ATP was monitored by SDS-PAGE and autoradiography. Samples for mass spectrometry analysis were prepared identically, but with non-labelled ATP.

### *In silico* procedures

DNA and protein sequence analysis was performed using the MacVector^®^ software (versions 12 to 16). Numerical data were analysed using the Prism^®^ software (version 5). Greyscale and colour images were optimised for contrast using Photoshop^®^ CS3 (Adobe). Composite figures were assembled using the Intaglio^®^ software (Purgatory). For the length measurements of cells, the digital images were imported into the ImageJ 1.42q software (Wayne Rasband, National Institute for Health, USA) analysed using the measurement line tool. Measurements in centimetres were then normalised using the integrated size bars. For the analysis of the cell body lengths 50 randomly selected cells were measured. For the analysis of the cell body widths and flagella length 25 cells were analysed. Significance was determined using the two-sided U-test^104^. All greyscale images were cropped from contiguous parts of the original image; no recombination of lanes was performed. Digital enhancements, using Adobe Photoshop CS3, were performed over the entire greyscale images and restricted to tonality (curve) optimisation and size adjustments.

## Supplementary information

Supplementary Information

## Data Availability

All data and materials generated for this study may be obtained from the corresponding author upon written request with the stipulation that any work derived from the data and materials will cite the source.
