# Supplementary material for: Leishmania donovani 90 kD Heat Shock Protein – Impact of Phosphosites on Parasite Fitness, Infectivity and Casein Kinase Affinity
Source: Sci Rep. 2019 Mar 25;9:5074. doi: 10.1038/s41598-019-41640-0 (PMC6434042; doi:10.1038/s41598-019-41640-0)
Supplement: Supplementary file 1 — Supplementary Information [file 41598_2019_41640_MOESM1_ESM.pdf]

Leishmania donovani 90 kD Heat Shock Protein – Impact of Phosphosites on Parasite Fitness, Infectivity and Casein Kinase Affinity

Supplementary Information

Antje Hombach-Barrigah[1], Katharina Bartsch[1], Despina Smirlis[2,4], Heidi Rosenqvist[3,6], Andrea MacDonald[1], Florent Dingli[5], Damarys Loew[5], Gerald F. Späth[2], Najma Rachidi[2], Martin Wiese[3], and Joachim Clos[1]*

[1]Bernhard Nocht Institute for Tropical Medicine, Hamburg, Germany

[2]Institut Pasteur and Institut National de Santé et Recherche Médicale INSERM U1201, Unité de Parasitologie Moléculaire et Signalisation, Paris, France

[3]Strathclyde Institute of Pharmacy and Biomedical Sciences (SIPBS) University of Strathclyde, Glasgow, Scotland, UK

[4] Hellenic Pasteur Institute, Athens, Greece

[5] Laboratoire de Spectrométrie de Masse Protéomique, Centre de Recherche, Institut Curie, PSL Research University, Paris, France

[6] current address: Novo Nordisk A/S, Gentofte, Denmark

*) corresponding author:

Joachim Clos, Bernhard Nocht Institute for Tropical Medicine, Hamburg, Germany

phone: +49 40 42818 481

e-mail: clos@bnitm.de

Key words: *Leishmania*, HSP90, casein kinase, protein phosphorylation, signal transduction, infectivity

Table S1. Primer sequences for the mutations of various HSP90 phosphorylation sites are listed in this table. The modified base triplets are highlighted in the sequence.

Mutation	Sequence
**T_{21}-I_{21}**	AA**CAT**CTTCTACTCGAACAAGGAG
**T_{21}-D_{21}**	AA**CGA**CTTCTACTCGAACAAGGAG
**T_{21}rev**	GATGATCAGCGACATCAAC
**T_{100}-I_{100}**	GG**CAT**CAAGGCGTTCATGGAGGCG
**T_{100}-D_{100}**	GG**CGA**CAAGGCGTTCATGGAGGCG
**T_{100}rev**	GGAGCGCGCGATCGTGCC
**T_{211}-A_{211}**	ACG**GCG**GAGAAGGAGGTGACGGACGAGGACGAG
**T_{211}-D_{211}**	ACG**GAC**GAGAAGGAGGTGACGGACGAGGACGAG
**T_{216}-A_{216}**	ACGACGGAGAAGGAGGTG**GCG**GACGAGGACGAG
**T_{216}-D_{216}**	ACGACGGAGAAGGAGGTG**GAC**GACGAGGACGAG
**T_{211}/T_{216}-A_{211}/A_{216}**	ACG**GCG**GAGAAGGAGGTG**GCG**GACGAGGACGAG
**T_{211}/T_{216}rev**	CTTCTCCACCATCAGCTCG
**T_{223}-A_{223}**	GAGGAT**GCG**AAGAAGGCCGCCGAGGAC
**T_{223}-D_{223}**	GAGGAT**GAC**AAGAAGGCCGCCGAGGAC
**T_{223}rev**	CTCGTCCTCGTCCGTCACCTC
**S_{289}-A_{289}**	CAT**CGC**CAACGACTGGGAGGAC
**S_{289}rev**	GCCTTGTAGAAGGCCGCG
**S_{526}-A_{526}**	TTCGAGGAG**GCG**GAGGAGGAGAAGCAGCAG
**S_{526}rev**	GTGCACGCCCTCCTTCGTCAG
**S_{594}-A_{594}**	GCGAC**GCC**AGCATGGCGCAGTACATGATG
**S_{595}-A_{595}**	GCGACTCC**GCC**ATGGCGCAGTACATGATG
**S_{594}/S_{595}-A_{594}/A_{595}**	GCGAC**GCCGCC**ATGGCGCAGTACATGATG
**S_{594}/S_{595}-D_{594}/D_{595}**	GCGAC**GGACGA**CATGGCGCAGTACATGATG
**S_{594}/S_{595}rev**	GCAGCGCCTGGTTGCGCATG
**T_{693}-A_{693}**	CGG**CGC**CTCCAGCATGGAGCAGGTGGAC
**T_{693}-D_{693}**	CGG**CGA**CTCCAGCATGGAGCAGGTGGAC
**S_{694}-A_{694}**	CGGCAC**CGC**CAGCATGGAGCAGGTGGAC
**S_{694}-D_{694}**	CGGCAC**CGA**CAGCATGGAGCAGGTGGAC
**T_{693}/S_{694}rev**	GCGGTGACCTCCGCGGGGGCCG

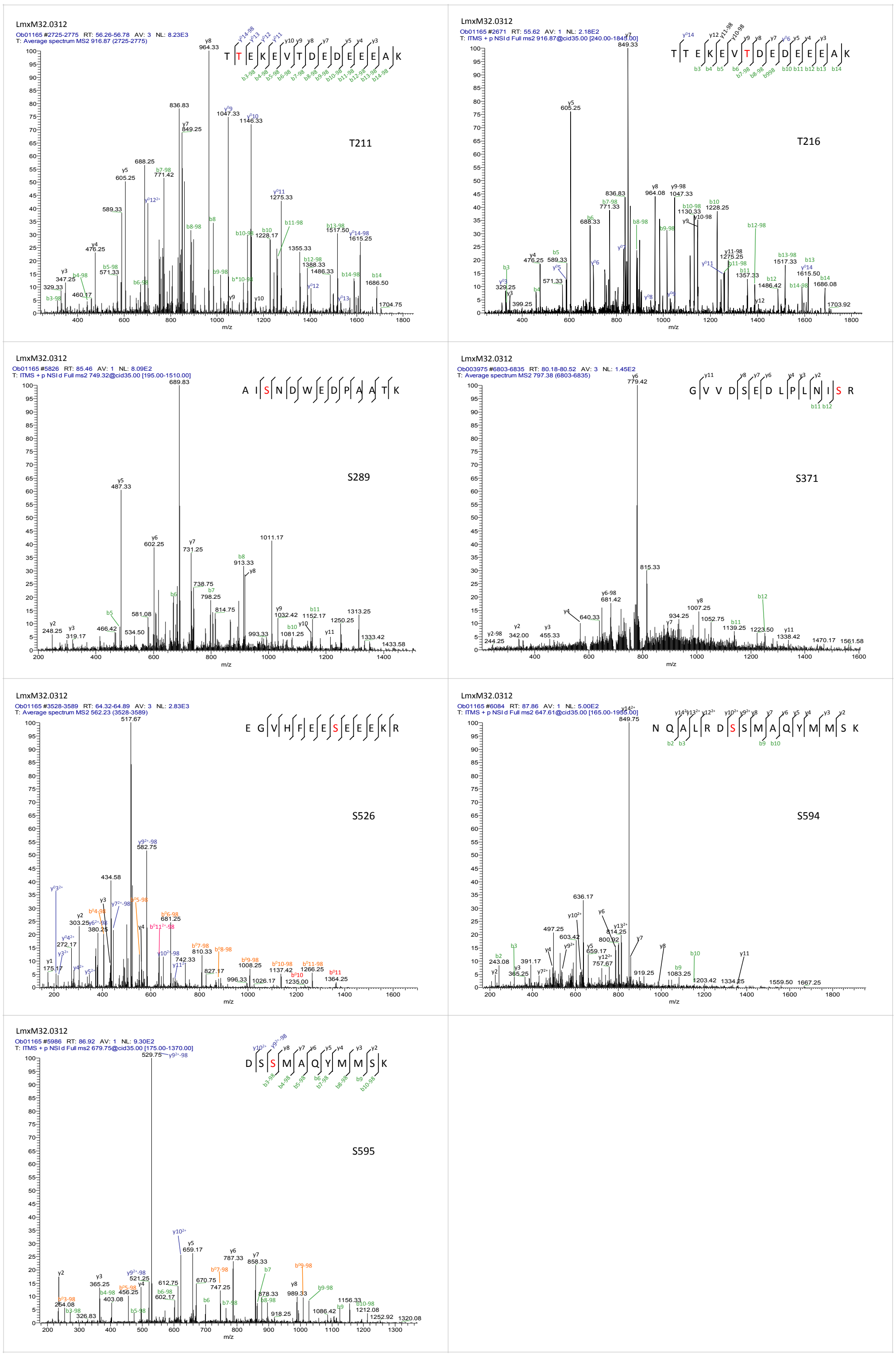

Figure S1: Mass spectra identifying phosphorylated peptides derived from the *Leishmania mexicana* HSP90

	- RAD	+ RAD
**HSP90rr**	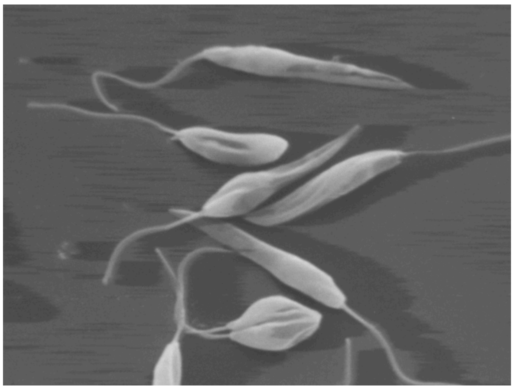	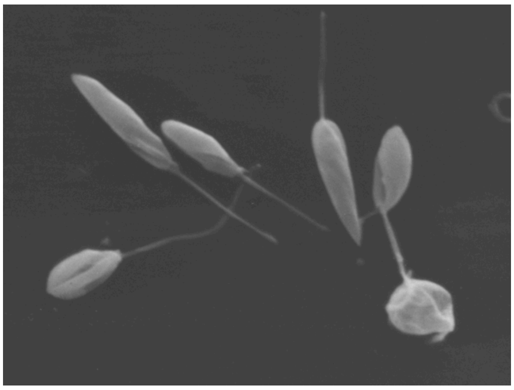
**HSP90wt**	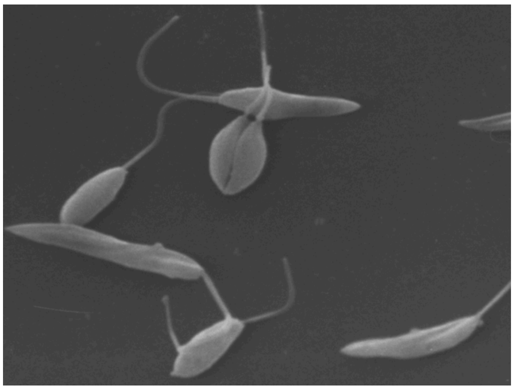	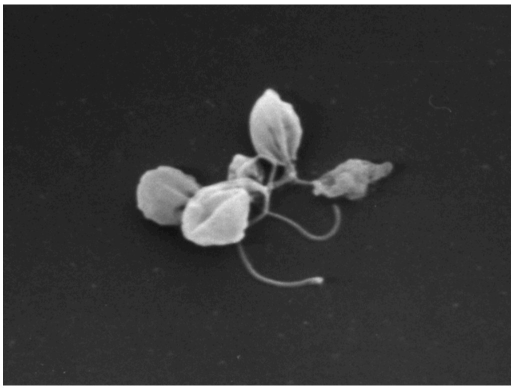
**P-site**	Ala +RAD	Asp +RAD
**T_{211}/T_{216}**	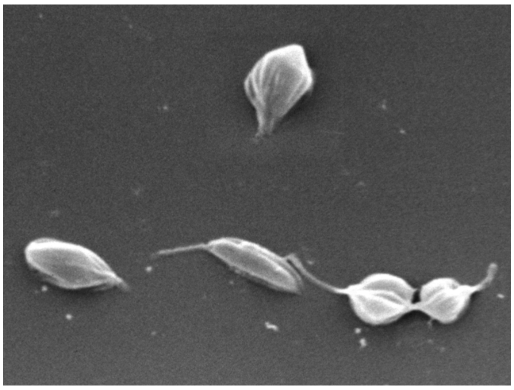	**n.a.**
**T_{223}**	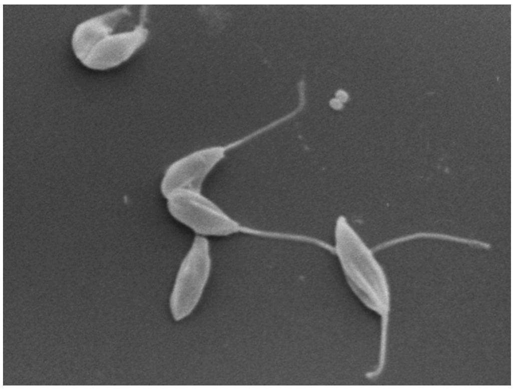	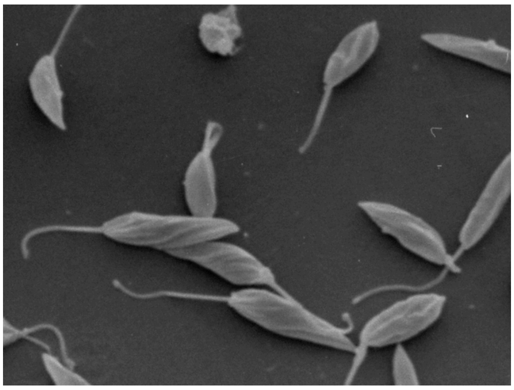

**S_{289}**	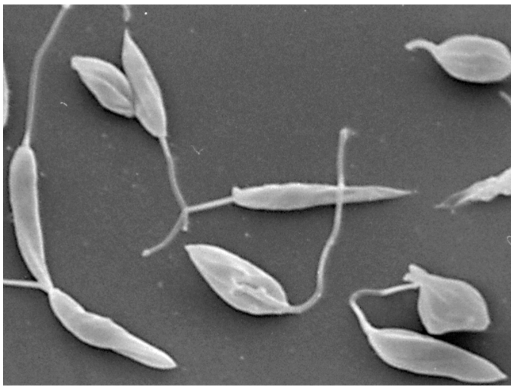	**n.a.**
**S_{526}**	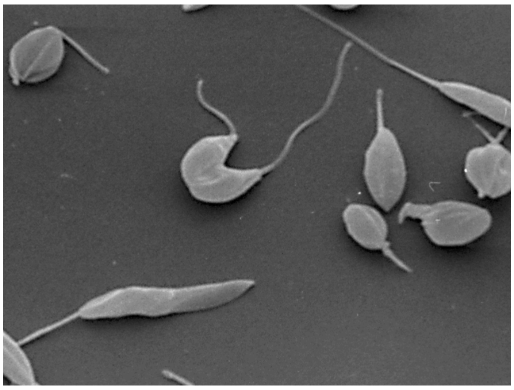	**n.a.**

Figure S2A: Representative images from the morphological analysis summarised in Fig. 3A. Scanning electron micrographs of *L. donovani*, ectopically expressing HSP90rr, HSP90wt, and P-site mutations of HSP90rr (serine or threonine to alanine or aspartic acid) and exposed to RAD at IC_{50}.

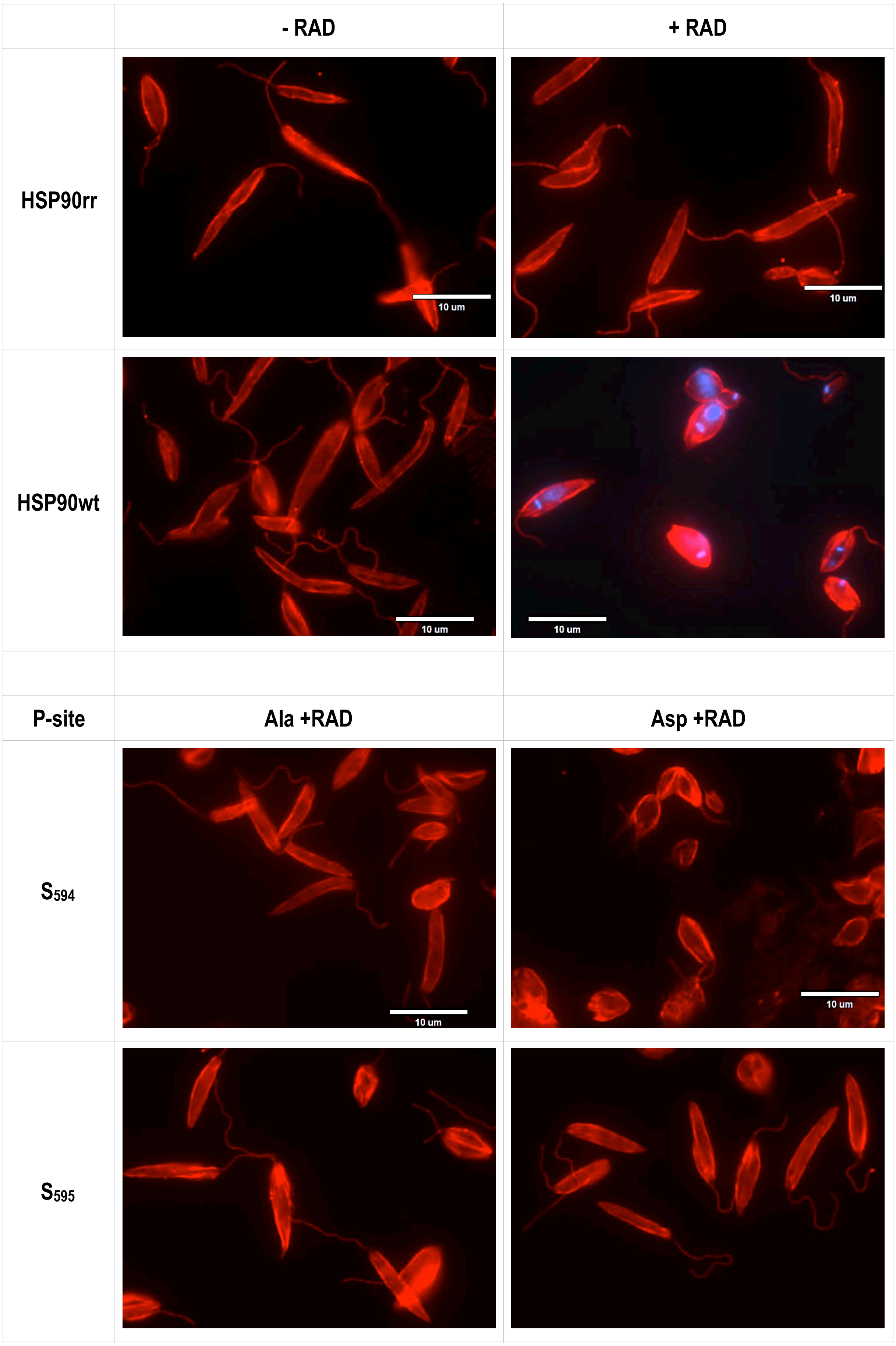

- RAD
+ RAD
HSP90rr
HSP90wt
P-site
Ala +RAD
Asp +RAD
S594
S595
10 um

T_{693}

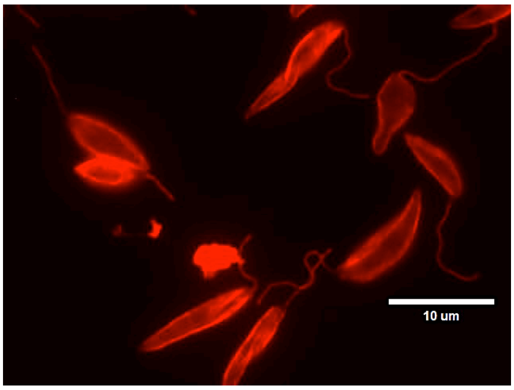

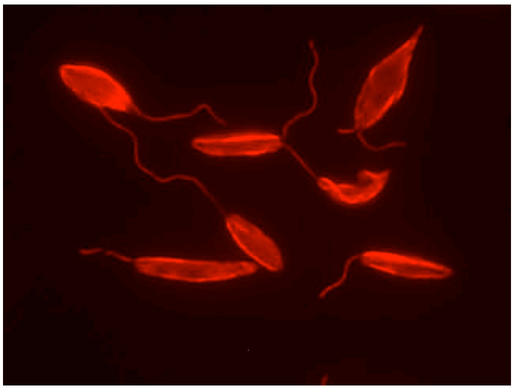

S_{694}

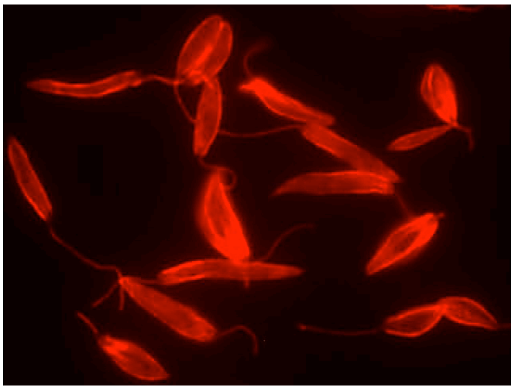

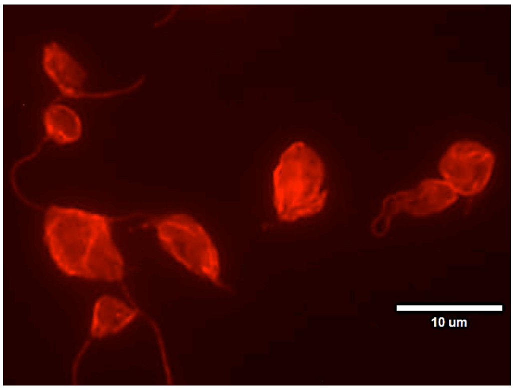

Figure S2B: Representative images from the morphological analysis summarised in Fig. 3B. Anti-tubulin indirect immune micrographs of *L. donovani*, ectopically expressing HSP90rr, HSP90wt, and P-site mutations of HSP90rr (serine or threonine to alanine or aspartic acid) and exposed to RAD at IC_{50}. One image (HSP90wt + RAD) shows DAPI co-costaining.
